# Saps1–3 Antigens in *Candida albicans*: Differential Modulation Following Exposure to Soluble Proteins, Mammalian Cells, and Infection in Mice

**DOI:** 10.3390/idr16040043

**Published:** 2024-06-28

**Authors:** Pedro F. Barbosa, Diego S. Gonçalves, Lívia S. Ramos, Thaís P. Mello, Lys A. Braga-Silva, Marcia R. Pinto, Carlos P. Taborda, Marta H. Branquinha, André L. S. Santos

**Affiliations:** 1 Laboratório de Estudos Avançados de Microrganismos Emergentes e Resistentes (LEAMER), Departamento de Microbiologia Geral, Instituto de Microbiologia Paulo de Góes (IMPG), Universidade Federal do Rio de Janeiro (UFRJ), Rio de Janeiro 21941-901, Brazil; pedrofdbarbosa@gmail.com (P.F.B.); fusariumsp@gmail.com (D.S.G.); liviaramos2@yahoo.com.br (L.S.R.); thaispdmello@gmail.com (T.P.M.); lysmonica@yahoo.com.br (L.A.B.-S.); 2 Programa de Pós-Graduação em Bioquímica (PPGBq), Instituto de Química (IQ), Universidade Federal do Rio de Janeiro (UFRJ), Rio de Janeiro 21941-909, Brazil; 3 Departamento de Microbiologia e Parasitologia, Instituto Biomédico, Universidade Federal Fluminense (UFF), Niterói 24210-130, Brazil; mpinto@id.uff.br; 4 Departamento de Microbiologia, Instituto de Ciências Biomédicas, Universidade de São Paulo (USP), São Paulo 05508-060, Brazil; taborda@usp.br; 5 Rede Micologia RJ—Fundação de Amparo à Pesquisa do Estado do Rio de Janeiro (FAPERJ), Rio de Janeiro 21941-901, Brazil

**Keywords:** *Candida albicans*, secreted aspartic proteases, Saps’ induction, organic source of nitrogen, cellular interaction, infection

## Abstract

The secreted aspartic peptidases (Saps) of *Candida albicans* play crucial roles in various steps of fungal–host interactions. Using a flow cytometry approach, this study investigated the expression of Saps1–3 antigens after (i) incubation with soluble proteins, (ii) interaction with mammalian cells, and (iii) infection in immunosuppressed BALB/c mice. Supplementation strategies involving increasing concentrations of bovine serum albumin (BSA) added to yeast carbon base (YCB) medium as the sole nitrogenous source revealed a positive and significant correlation between BSA concentration and both the growth rate and the percentage of fluorescent cells (%FC) labeled with anti-Saps1–3 antibodies. Supplementing the YCB medium with various soluble proteins significantly modulated the expression of Saps1–3 antigens in *C. albicans*. Specifically, immunoglobulin G, gelatin, and total bovine/human sera significantly reduced the %FC, while laminin, human serum albumin, fibrinogen, hemoglobin, and mucin considerably increased the %FC compared to BSA. Furthermore, co-cultivating *C. albicans* yeasts with either live epithelial or macrophage cells induced the expression of Saps1–3 antigens in 78% (mean fluorescence intensity [MFI] = 152.1) and 82.7% (MFI = 178.2) of the yeast cells, respectively, compared to BSA, which resulted in 29.3% fluorescent cells (MFI = 50.9). Lastly, the yeasts recovered from the kidneys of infected immunosuppressed mice demonstrated a 4.8-fold increase in the production of Saps1–3 antigens (MFI = 246.6) compared to BSA, with 95.5% of yeasts labeled with anti-Saps1–3 antibodies. Altogether, these results demonstrated the positive modulation of Saps’ expression in *C. albicans* by various key host proteinaceous components, as well as by in vitro and in vivo host challenges.

## 1. Introduction

Fungal infections have become increasingly significant in recent years due to a notable rise in their global incidence. This increase in fungal diseases may be influenced by various factors, including changes in the pathogens themselves, such as the emergence of new resistance and virulence attributes, environmental disturbances like global warming, and alterations in host health conditions. For instance, immunosuppressed individuals—often due to medical treatments such as chemotherapy, organ transplantation, or autoimmune diseases—face a unique challenge: their immune systems, the body’s defense against pathogens, are weakened or compromised. The host’s debilitated immunity significantly increases the susceptibility to various infectious agents [1,2].

The *Candida* genus stands as the most common cause of invasive fungal infections globally, with species within this genus acting as primary etiological agents of nosocomial fungal infections in hospital settings [3]. Fungal opportunistic pathogens belonging to the *Candida* genus have the capability of causing a wide spectrum of pathologies, spanning from superficial to invasive infections. Among these, the latter presents the most significant challenge due to its severity and the complexities associated with treatment, particularly in immunosuppressed individuals with compromised immune systems [4]. Among *Candida* species, *C. albicans* retains its status as the primary etiological agent predominantly associated with cases of invasive candidiasis in healthcare settings worldwide. This is largely attributed to *C. albicans’* extraordinary ability to infect virtually every anatomical site of the human body, facilitated by its adaptable genome, which enables rapid metabolism changes to suit various and distinct physicochemical environments [5].

A myriad of molecules and structures are involved in fostering the successful development of an infectious process instigated by *Candida* spp. Notably, a major virulence attribute of *C. albicans* is its ability to secrete a diverse array of aspartic-type peptidases, collectively designated as Saps [6]. Beyond their simple role of digesting proteinaceous molecules for nutritional purposes and supporting growth and proliferation, Saps also play other functions in the pathogenicity of *C. albicans*, including the (i) degradation of tissue components (e.g., extracellular matrix/basal membrane and surface membrane proteins) during invasion; (ii) evasion of the host immune system by cleaving different classes of immunoglobulins, complement proteins, proteinaceous peptidase inhibitors, cytokines, and antimicrobial peptides; and (iii) adherence to both abiotic (e.g., medical devices like catheters, prosthetic valves, artificial dentures, and many others) and biotic (e.g., epithelial cells and tissues) surfaces [7,8,9].

Saps are encoded by a family of 10 genes (*SAPs1-10*), with *SAP2* gene being one of the most extensively studied members. Sap2 has been shown to contribute to host tissue invasion by digesting or destroying cell membranes [10]. Notably, the use of *SAP*-deficient mutants has revealed that the *SAPs1*–*3* gene subfamily plays a role in tissue damage in both oral and vaginal reconstituted human epithelium (RHE) models, indicating their involvement in establishing *C. albicans* infections at mucosal surfaces [11,12]. Furthermore, it is well-known that Saps’ expression can be modulated by different physicochemical and biological parameters encountered in the environment colonized and/or infected by *C. albicans* [13]. For instance, the cultivation of different *C. albicans* strains in a chemically defined medium supplemented with single high-molecular-mass proteins as the sole nitrogen source, such as albumin, has been shown to induce the production and secretion of Saps into the extracellular environment [14,15,16].

Considering the multifunctional role of Saps in many virulence aspects of *Candida* spp., in the present work we aimed to investigate the expression of Saps1–3 antigens in *C. albicans* by a flow cytometry approach after growth in a chemically defined medium supplemented with different sources of soluble proteinaceous molecules, including sole (human and bovine serum albumins, immunoglobulin G, gelatin, fibrinogen, hemoglobin, mucin, and laminin) and mixed (human and fetal bovine sera) proteins. Additionally, we examined the expression of Saps1–3 after in vitro co-cultivation with either human (HEp-2) or murine (peritoneal macrophage) cells, and following in vivo challenges using an immunosuppressed murine model of disseminated fungal infection.

## 2. Materials and Methods

### 2.1. Microorganism and Growth Conditions

*Candida albicans* strain 11, which was isolated from the blood of a 46-year-old male [17], was used in the present study. This clinical strain is noteworthy for exhibiting the trailing phenomenon in terms of fluconazole and itraconazole susceptibility, along with the ability to express elevated levels of both surface-bound and extracellularly released Saps1–3, as previously documented by our research team [12,17]. Yeast cells were cultured into 1.2% yeast carbon base (YCB) medium (HiMedia Laboratories Ltd., Mumbai, India) supplemented with 0.1% bovine serum albumin (BSA; Sigma-Aldrich, St. Louis, MO, USA), pH 5.0, at 37 °C for 48 h (exponential growth phase) under slight agitation (100 rpm), which are well-known Sap-inducing conditions [17,18,19]. Cell growth was estimated by counting the number of yeast cells in a Neubauer chamber.

Cell viability was evaluated using the passive incorporation of propidium iodide (PI) staining, a nucleotide base intercalator. The fungal cells were incubated with PI (1 mg/mL) for 10 min in phosphate-buffered saline (PBS) at room temperature, protected from light. The cells were then washed in PBS, harvested at 500× *g* for 5 min, dispensed into 96-well opaque plates, and immediately analyzed using an LSRFortessa flow cytometer (BD Bioscience, Milpitas, CA, USA) with excitation and emission wavelengths of 540 and 608 nm, respectively. Yeasts permeabilized with 4% paraformaldehyde served as non-viable cells (positive staining). The results were expressed as the percentage of red-labeled fluorescent cells.

### 2.2. Effect of BSA Concentration on the Expression of Saps1–3 Antigens

*Candida albicans* yeasts were cultivated in YCB medium supplemented with different concentrations of BSA (0.1, 0.05, 0.01, 0.005, and 0.001%) for 48 h at 37 °C under slight agitation. Subsequently, fungal cells (10^6^ yeasts) were washed in PBS and processed for flow cytometry to evaluate the expression of Saps1–3 antigens as described below.

### 2.3. Effect of Different Soluble Proteins on the Expression of Saps1–3 Antigens

In this set of experiments, *C. albicans* yeasts were grown in YCB medium supplemented with 0.05% from each of the following proteinaceous sources: BSA, human serum albumin (HSA), fetal bovine serum (FBS), human serum, human immunoglobulin G (IgG), human hemoglobin, porcine gelatin, porcine mucin, human plasmatic fibrinogen, and human laminin. All the proteins used in this study were purchased from Sigma-Aldrich (St. Louis, MO, USA). Subsequently, fungal cells (10^6^ yeasts) were washed in PBS and processed by flow cytometry to evaluate the expression of Saps1–3 antigens as described below.

### 2.4. Effect of the Interaction between Yeasts and Mammalian Cells on the Expression of Saps1–3 Antigens

#### 2.4.1. Epithelial Cells

HEp-2 cell lineage (human laryngeal carcinoma epithelial cells) was maintained and grown to confluence in 25 cm^2^ culture flasks containing Dulbecco’s modified Eagle’s medium (DMEM; Sigma-Aldrich, St. Louis, MO, USA) supplemented with 10% FBS at 37 °C with 5% CO_2_. The pH was maintained at 7.2 by the addition of 3 g/L of HEPES and 0.2 g/L of NaHCO_3_ to the medium. Epithelial cells, initial inoculum 5 × 10^4^ cells/mL, were subcultured every 2 days and maintained in log phase.

#### 2.4.2. Macrophage Cells

Peritoneal macrophages were isolated from BALB/c mice following the injection of 2.5 mL of Brewer thioglycolate medium at 3% concentration (Thermo-Fisher, Waltham, MA, USA) into the peritoneum. After 3–5 days post-stimulation, the animal was euthanized. A small incision was made in the lower abdomen using sterile scissors to insert a needle containing 5 mL of sterile PBS into the peritoneal cavity, and the solution was aspirated to collect the peritoneal cells. The isolated macrophages were then cultured in 25 cm^2^ culture flasks containing DMEM supplemented with 10% FBS and 1% penicillin/streptomycin solution (Sigma-Aldrich, St. Louis, MO, USA) [20,21].

#### 2.4.3. Interaction Assay

Mammalian cells were plated onto 24-well dishes at a density of 10^5^ cells per well and incubated at 37 °C for 24 h in DMEM supplemented with 10% FBS. Then, mammalian cells were washed twice with PBS and finally rinsed in DMEM. Fungal suspensions were prepared in DMEM to generate a ratio of 10 yeasts per mammalian cell. Interactions between fungal and mammalian cells occurred at 37 °C with 5% CO_2_ for 2 h. Subsequently, the interaction systems were washed three times with PBS to remove non-adherent yeasts, followed by the addition of 1 mL of cold sterile water to each well in order to lyse the mammalian cells and recover *C. albicans* yeasts. Two controls were prepared: (i) wells without mammalian cells were used to support yeasts’ growth in DMEM medium and (ii) wells containing uninfected mammalian cells. Finally, the recovered yeasts were used to quantify Saps1–3 antigens by flow cytometry as described below.

### 2.5. Effect of Murine Infection on the Expression of Saps1–3

Isogenic female BALB/c mice (6–8 weeks old) were bred at the University of São Paulo (USP; São Paulo, SP, Brazil) in an animal facility under specific pathogen-free conditions, with a constant temperature, 12h light/dark cycles, ad libitum feeding, and oversight by a single individual trained and specialized in laboratory animal care. The procedures involving mice and their care were conducted according to the local ethics committee and international rules. All experiments were approved by the Institutional Animal Care and Use Committee of Institute of Biomedical Sciences (ICB) of USP (number 042-127-02). For the experiment, mice were immunosuppressed by the administration of doses of 100 mg/kg of cyclophosphamide intraperitoneally 4 days and 1 day prior to infection with *C. albicans*. The mice were kept in cages lined with wood shavings and closed with an autoclaved filter, and served autoclaved food and water in order to maintain a sterile environment. Three immunosuppressed female BALB/c mice were intravenously infected with 10^5^ yeasts of *C. albicans*. The mice that survived after 5 days post-infection were anaesthetized and then euthanized by cervical dislocation. Then, the kidneys were dissected aseptically, weighed, homogenized in 1 mL of sterile PBS, and spread onto BHI agar plates to isolate the fungal colonies. Finally, ten yeast colonies were randomly selected from the BHI plate, washed in PBS, and grown in YCB-BSA medium at 37 °C for 48 h under agitation (100 rpm) to quantify Saps1–3 antigens by flow cytometry as described below.

### 2.6. Evaluation of Saps1–3 Antigens

In all the experiments, *C. albicans* (10^6^ yeasts) were fixed at 4 °C in 4% paraformaldehyde in PBS, pH 7.2, for 20 min, followed by extensive washing in the same buffer. The fixed cells maintained their morphological integrity, as observed by light microscope inspection. Then, the fungal cells were incubated for 1 h at 25 °C with a 1:250 dilution of rabbit anti-Saps1–3 antibodies (kindly provided by Dr. Nina Agabian, University of California, Los Angeles, CA, USA), and later incubated for an additional hour with a 1:200 dilution of fluorescein isothiocyanate (FITC)-labeled goat anti-rabbit IgG. For flow cytometry analysis, fungal cells were examined in an FACSCan flow cytometer (BD^®^, Bioscience, La Jolla, CA, USA) equipped with a 15mW argon laser emitting at 488 nm. Unlabeled cells and those labeled only with the secondary antibody were used as controls. For all the systems, the autofluorescence of cells was also measured. Each experimental population was mapped by using a two-parameter histogram of forward-angle light scatter versus side scatter. The mapped population (*n* = 10,000) was then analyzed for log green fluorescence by using a single-parameter histogram [17]. The results were expressed as the percentage of fluorescent cells (%FC) and mean of fluorescence intensity (MFI).

### 2.7. Statistical Analysis

All experiments were performed at least three times in triplicate. The results were statistically analyzed by one-way analysis of variance (one-way ANOVA). The correlation tests were determined by Pearson’s correlation coefficient (*r*). *p* values less than or equal to 0.05 were considered significant. All analyses were performed using the GraphPad Prism program.

## 3. Results and Discussion

### 3.1. Expression of Saps1–3 in C. albicans Yeasts Is Dependent on BSA Concentration

**Figure 1:**
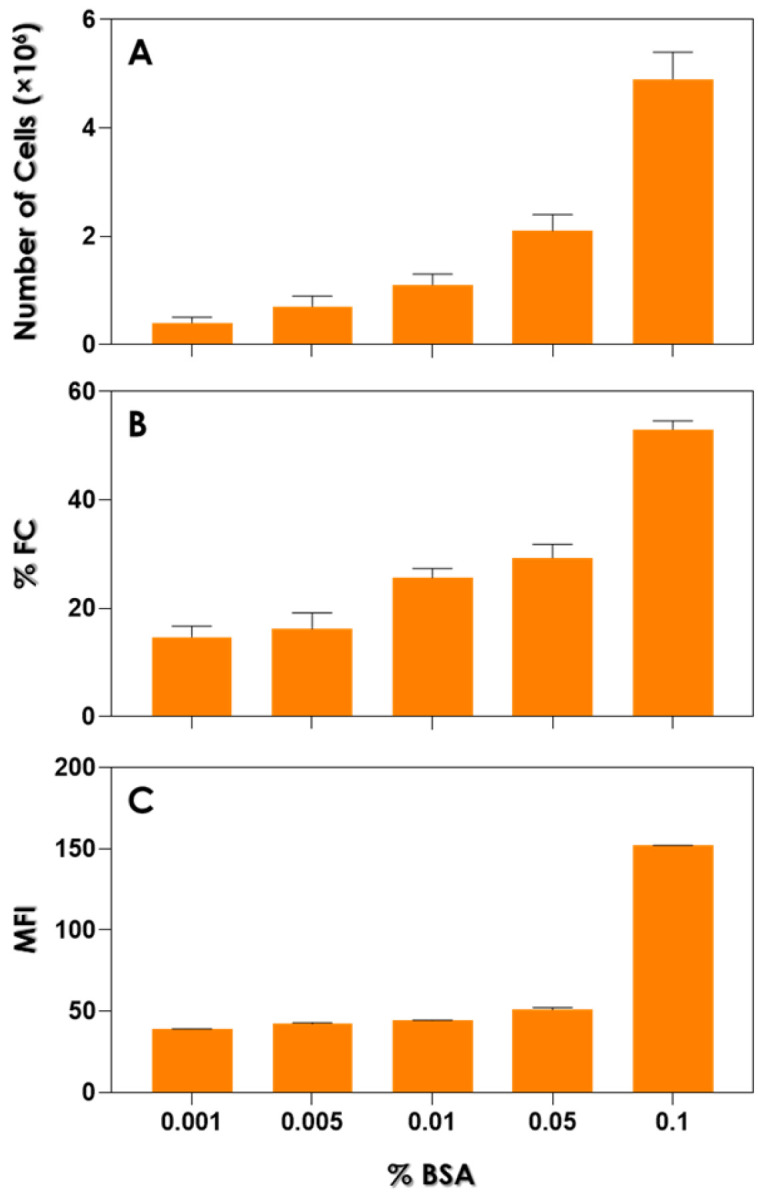
Effects of different concentrations of BSA on the expression of Saps1–3 antigens in *Candida albicans* yeast cells. Yeasts were incubated in YCB medium supplemented with 0.1, 0.05, 0.01, 0.005, and 0.001% BSA at 37 °C for 48 h and cell growth was estimated by counting yeast cells in a Neubauer chamber (**A**). Yeasts collected after 48 h of incubation in YCB medium supplemented with different concentrations of BSA were fixed with 4% paraformaldehyde and incubated with the primary anti-Saps1–3 antibodies from *C. albicans* and then with FITC-labeled secondary antibody. The results were analyzed by flow cytometry and expressed as (**B**) the percentage of fluorescent cells (%FC) and (**C**) mean of fluorescence intensity (MFI) values. Values represent the mean ± standard deviation of three independent experiments.

Initially, we assessed the growth of *C. albicans* when cultured in YCB medium containing various concentrations of BSA to determine its impact on the number of yeast cells and the expression of Saps1–3 antigens (Figure 1). Our results demonstrated a direct association between cell growth and the concentration of BSA used to supplement the YCB medium (Figure 1A). Consequently, a higher number of cells was observed in the growth medium containing 0.1% BSA (mean of 4.9 × 10^6^ yeasts), while a reduced growth occurred with BSA at 0.001% (mean of 0.4 × 10^6^ yeasts). A similar trend was observed considering the expression of Saps1–3 antigens in *C. albicans* yeasts (Figure 1B). In this context, when comparing variations from the highest to the lowest BSA concentrations added to YCB medium, our results showed a %FC ranging from 53 ± 1.6 to 14.7 ± 2.0 (Figure 1B), as well as the MFI varying from 152 ± 0.1 to 14.7 ± 2.0, respectively (Figure 1C). This further supports the idea that the induction of expression of Saps1–3 in *C. albicans* yeasts depends on the availability of the nitrogen source present in the environment.

**Figure 2:**
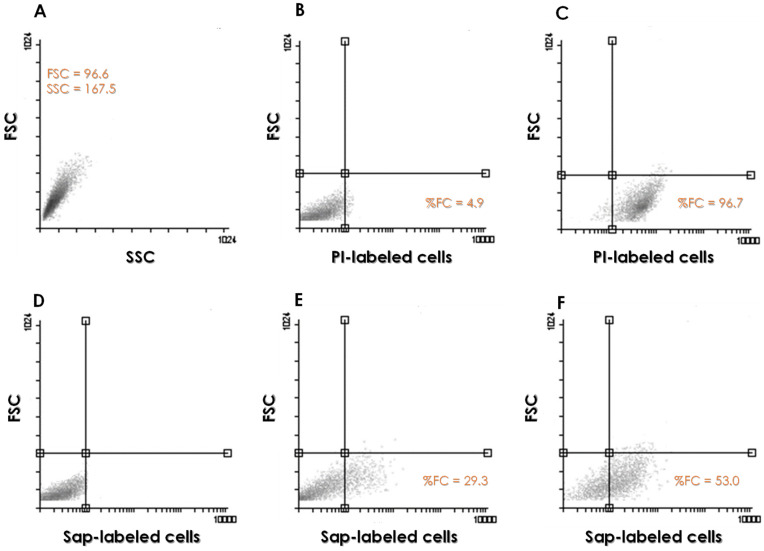
Representative flow cytometry histograms of *C. albicans* cells under different growth conditions. (**A**) Morphometric parameters, size (FSC), and granularity (SSC) of the fungal cells grown in YCB supplemented with 0.05% BSA. No differences were observed in these morphometric parameters when *C. albicans* cells were grown in YCB supplemented with different BSA concentrations used in this study. Viability of fungal cells grown in YCB-BSA (0.05%) was assessed by passive incorporation of propidium iodide in untreated (**B**) and 4% paraformaldehyde-treated cells (**C**). Detection of Sap1–3 antigens in *C. albicans* cells cultured in YCB supplemented with BSA at both 0.05% (**E**) and 0.1% (**F**). Yeast cells labeled only with the second antibody (positive control) are shown in (**D**).

The morphometric parameters of *C. albicans* yeasts grown in YCB-BSA medium, including size and granularity, were analyzed, revealing a homogeneous cell population (Figure 2A). The viability of the analyzed cell population was evaluated through passive incorporation of PI. As expected, the 48-h-old fungal population exhibited an insignificant number of dead cells (less than 5% of the total population) (Figure 2B), compared to the formaldehyde-fixed cells, where more than 95% of the population was labeled by PI (Figure 2C). Additionally, representative histograms were presented to illustrate the Sap1–3-labeled cell populations grown in YCB medium supplemented with BSA at concentrations of 0.05% (Figure 2E) and 0.1% (Figure 2F).

**Figure 3:**
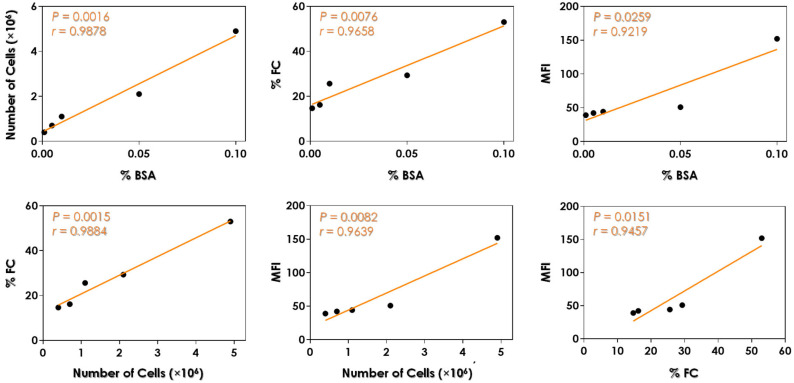
Pearson correlation analysis between the parameters evaluated in this study. Positive (*r* value) and significant (*p* value) correlations were observed among the percentage of BSA added in the YCB medium in relation to both percentage of Sap1–3-labeled cells (%FC) and mean of fluorescence intensity (MFI) of yeast cells. Similar trend was observed for the correlations between number of cells and %FC, number of cells and MFI, and MFI and %FC.

Notably, the supplementation strategies involving increasing concentrations of BSA as the sole nitrogen source in the YCB medium revealed a positive and significant (*p* < 0.05) correlation among BSA concentration and the *C. albicans* growth rate, the %FC labeled with anti-Saps1–3 antibodies, and the quantity of Saps1–3 antigens as suggested by the MFI parameter (Figure 3). Similarly, positive and significant correlations were also observed between the following parameters: number of cells and %FC, number of cells and MFI, and %FC and MFI (Figure 3).

Our findings parallel those reported in the available literature, which demonstrates that BSA serves as an effective inducer for *SAP* gene expression in various *Candida* species, including *C. albicans*, *C. tropicalis*, *C. parapsilosis*, and *C. haemulonii*, as well as other medically significant fungal species such as *Phialophora verrucosa* and *Fonsecaea pedrosoi* [22,23,24,25,26,27,28]. Indeed, it has been previously documented that the synthesis and secretion of Sap2 by *C. albicans* cells into a defined medium can be regulated by exogenous proteins (such as albumin and hemoglobin) employed as the sole nitrogen source through a positive feedback mechanism. In this mechanism, the accumulation of peptides resulting from the proteolysis of high-molecular-mass proteins leads to the induction of this peptidase [29,30].

Nitrogen is a crucial nutrient for microbial growth, and a significant strategy for maximizing peptidase production induction involves limiting the supply of inorganic nitrogen [31]. In light of this, the development of a chemically defined nitrogen-limited growth medium has been employed to assess the capacity of various molecules to stimulate *Candida* peptidase production. The impact of amino acid supplementation on Saps production in *Candida* yeasts was previously assessed through Western blot experiments. Notably, the addition of free amino acid mixtures in the culture medium as a nitrogen source, equivalent to the composition of BSA, did not induce Sap production [32]. These results underscore that peptidases are induced in the culture medium when the nitrogen source is defined by the presence of peptides/proteins rather than amino acids. Similarly, modulations in medium pH also influence *Candida* cells, impacting plasma membrane function, nutrient availability, and enzymatic activity [33]. In this context, the synthesis of Saps can be directly influenced by the pH of the growth medium, given that these enzymes exhibit optimal activity at acidic pH [34]. Immunoblotting studies on *C. albicans* strains identified pH 4.0 as optimal in the Sap induction medium supplemented with BSA, while pH 5.0 was determined as optimal for the same Sap induction medium without BSA [35]. This pH discrepancy was attributed to more active cell growth at pH 4.0 in the presence of BSA. The enhanced growth is explained by the nitrogen source supplied through the degradation of BSA, which has an optimal pH range of 3.5–4.0 [36]. Furthermore, no induction was observed in a medium of neutral pH, irrespective of the presence of BSA, emphasizing that modulations in medium pH may also have the capability to either promote or inhibit Saps’ production.

In the context of infection, many pathogenic fungi must acquire nitrogen from a broad range of proteinaceous molecules present in different anatomical sites inside the host [37]. The cleavage and uptake of available nitrogen sources in the host’s organism are highly associated with Saps’ production, making it essential for the survival and growth of *Candida* species [38].

### 3.2. Soluble Proteins Differently Modulate the Expression of Saps1–3 in C. albicans Yeasts

A wide array of macromolecules, particularly soluble proteins, is capable of inducing the secretion of peptidases in *Candida* species. In view of this, various research groups have successfully analyzed the secretion of Saps by cultivating *C. albicans* strains in a chemically defined medium supplemented with distinct soluble proteins, including BSA, keratin, ovalbumin, laminin, hemoglobin, lactoferrin, and human serum. Saps’ secretion was predominantly validated through Western blot and agarose spot assays [39,40,41,42,43,44]. In this set of experiments, we evaluated the ability of different soluble proteinaceous sources (either purified or mixed proteins) to modulate the expression of Saps1–3 antigens in *C. albicans*. For this purpose, yeast cells were cultivated in a chemically defined medium supplemented with serum proteins (albumin, fibrinogen, hemoglobin, IgG), extracellular matrix proteins (laminin, mucin and gelatin, representing hydrolyzed collagen), as well as complex protein mixtures (total human and bovine sera) and then analyzed by flow cytometry using anti-Saps1–3 antibodies.

Our results showed that IgG, total fetal bovine serum (FBS), total human serum, and gelatin significantly reduced the number of Saps1–3-expressing cells in the fungal population compared to yeasts grown in the presence of BSA molecules. In contrast, laminin, HSA, fibrinogen, hemoglobin, and mucin considerably increased the percentage of yeasts labeled with anti-Saps1–3 antibodies compared to BSA (Table 1). Moreover, the amount of Saps by Saps1–3-positive *C. albicans* cells was evidenced by the MFI parameter. Interestingly, the soluble purified proteins gelatin, HSA, hemoglobin, and mucin were able to induce an augmentation in the expression of Saps1–3 antigens compared to BSA stimulation. However, the complex mix of proteins found in both bovine and human sera reduced the production of Saps1–3 antigens by *C. albicans* yeasts (Table 1).

Our findings are aligned with the existing literature, as it is known that when *C. albicans* cells invade host tissues, they must surpass surface barriers like skin and mucosa [45]. In this context, Saps aid in penetration by cleaving extracellular matrix proteins such as laminin, fibronectin, collagen, and mucin [46,47]. Studies have also shown that *C. albicans* strains can both adhere to and enzymatically degrade mucins in oral cavities and the gastrointestinal tract, thereby modulating *Candida* populations [48,49]. Although peptidase production is induced by various protein substrates, the specific molecular mechanisms regulating peptidase induction remain unknown. One hypothesis for inducing peptidase synthesis and secretion involves transmembrane signal transduction, wherein cell surface receptors bind proteinaceous ligands, signaling and inducing peptidase synthesis. Recent studies have implicated mucins in cellular signaling. Mucins act through substantially different mechanisms; MUC4 acts as a receptor ligand and MUC1 acts as a docking protein for signaling molecules. Both molecules provide steric protection to epithelial surfaces [50]. Interestingly, supplementation strategies with gelatin, which corresponds to the denatured form of type I collagen, a glycoprotein considered as one of the main targets for the adhesion of *Candida* species [51], reduced the number of Saps1–3-positive cells. Nonetheless, a compensation phenomenon was observed, as these fungal cells were able to produce 1.75 times more Saps1–3 antigens when compared to those grown in a BSA-containing medium. A similar compensatory strategy was observed when *C. albicans* cells were incubated with human IgG. These results may suggest distinct biological regulations promoted by soluble proteins in *C. albicans* cells, as the secretion of Saps is a key mechanism for evading the host’s immune system by *Candida* species [52]. Furthermore, Saps play a role in the progression of candidiasis by aiding *Candida* yeasts in degrading hemoglobin, which is a crucial event for acquiring iron, facilitating yeast cell proliferation during systemic infection [53]. Noticeably, the clinical isolate used in the present study exhibited significant Saps1–3 expression when grown in media supplemented with hemoglobin and HSA as the sole nitrogen source, potentially linked to its bloodstream isolation.

Human and animal sera are complex media composed of different molecules such as proteins, lipids, and immune cells [54]. Therefore, the interaction of *C. albicans* with serum has been a long-term focus in the field of medical mycology. Although many studies in the literature have demonstrated that human serum can stimulate *C. albicans* biofilm growth, increase the number of planktonic cells, and upregulate the expression of virulence genes [55], we observed contrary effects to this pattern. In fact, some inhibitory events in *Candida* physiology have been associated with components of the innate immune system present in both bovine and human sera [56].

Previous studies using systemic infection models in mice have shown that *C. albicans* cells can be rapidly cleared from the bloodstream, correlating this event with the ability of human serum to inhibit yeast adhesion on the surface of endothelial cells [57,58]. Furthermore, innate immunity proteins, constituents of mammalian serum, play several roles against microbial infections [59]. Mammalian serum amyloid A (SAA) is a major acute phase protein that exhibits a massive increase in plasma concentration during *C. albicans* infection [60]. In this context, recombinant human and mouse SAA1 demonstrated potent antifungal activity against *C. albicans*, disrupting plasma membrane integrity and inducing rapid fungal cell death [61].

It is worth noting that there is a limited number of studies regarding the inhibitory effects of mammalian serum against *Candida* species, with results similar to those presented in the present study. Therefore, we emphasize the need for further investigations to assess whether such effects are restricted to the clinical strain studied or shared among other *Candida* species.

### 3.3. C. albicans–Mammalian Cells Interactions Induce the Expression of Saps1–3 Antigens

Since the interaction between *C. albicans* and host cells involves specific fungal recognition molecules [62], we aimed to investigate whether the expression of Saps1–3 antigens by *C. albicans* cells would be modulated upon interaction with distinct mammalian cells. Our results demonstrated that both epithelial and macrophage cells induced an increase in the expression of Saps1–3 antigens in the recovered *C. albicans* yeasts after the infectious process compared to yeasts cultured only in the YCB-BSA medium (Table 2). These results were evident through the augmentation in the number of Saps1–3-expressing *C. albicans* cells and the increased amount of these fungal antigens, as judged by the MFI parameter, after co-cultivation with both mammalian cells tested in the present study (Table 2).

*Candida albicans* exhibit selective adherence to buccal and vaginal epithelial cells in humans, which is believed to play a crucial role in the pathogenesis of mucocutaneous candidiasis [63,64,65]. Genomic studies demonstrated a progressive expression pattern of *SAP* genes during the stages of cutaneous infection, observing an order of *SAP1* and *SAP2* > *SAP8* > *SAP6* > *SAP3* in an in vitro model of cutaneous candidiasis based on reconstituted human epidermis [66]. This study indicated that the protective effect of the aspartic peptidase inhibitor, pepstatin A, during epidermal infection, along with an attenuated virulence phenotype of *SAP*-deficient mutants, is suggestive of a correlation between the observed expression of *SAP* genes and tissue damage in the skin [67].

*Candida*–macrophage interactions are important immune defense responses that can lead to disseminated candidiasis in humans [68]. Typically, the phagocytic process involves the engulfment of a microbe by the phagocyte followed by its killing. Saps contribute to the virulence of *C. albicans* by facilitating tissue invasion and aiding in the evasion of host immune responses. Notably, Sap antigens have been observed on the surface of fungal elements colonizing mucosa, penetrating tissues during disseminated infection, and evading macrophages even after the phagocytosis of *Candida* cells [68]. Herein, we observed a significant increase in the production of Saps1–3 by *C. albicans* cells after interaction with macrophages, potentially enhancing this fungus’s ability to interact with phagocytic cells (Table 2).

To successfully establish infections, *Candida* species have developed several strategies to evade the host immune system [69]. The human complement system is targeted by *C. albicans* Saps1–3 due to their ability to bind and cleave essential proteins such as C3b, C4b, and C5, inhibiting complement pathways and generating a micro-environment of reduced host innate immune functions as a consequence [70]. Moreover, similar approaches focused on *C. tropicalis*-secreted aspartic peptidase 1 (Sapt1) have shown that Sapt1 efficiently cleaved DC-SIGN, the receptor on antigen-presenting dendritic cells, as well as human mannose-binding lectin (MBL) and collectin-11, which are initiating molecules of the lectin pathway of the complement system [71]. Collectively, these data demonstrate how the production and secretion of Saps constitute one of the most relevant fungal strategies in the interaction with mammalian cells and in the evasion of host immunosurveillance processes.

### 3.4. In Vivo Mouse Infection Promotes Expression of Saps1–3 Antigens

Given that several members of the Sap family are expressed in vivo by *C. albicans* and are purported to play a significant role in the progression of candidiasis, our final objective was to analyze whether yeasts recovered from in vivo infections could exhibit alterations in the expression of Saps1–3 antigens, using immunosuppressed BALB/c mice as a model. Our results showed that *C. albicans* yeasts recovered from the kidneys of infected mice produced 4.8-fold more Saps1–3 antigens compared to the yeasts cultured in YCB-BSA medium (Table 2).

The observed increase in Saps’ expression during in vivo experimentation may suggest the cleavage of key host proteins during infection. Some of these potential target host proteins were tested in this study (Table 1). This correlation hypothesis could highlight the essential role of these enzymes in nutrient acquisition, in addition to facilitating the invasion and dissemination of the fungus in the host, aligning with data previously documented in the literature [72].

As previously mentioned, the virulence of *C. albicans* is a multifactorial event wherein various genes are expressed to produce distinct classes of molecules, such as Saps, collectively supporting the success of the infectious process [73,74]. The contribution of *SAP* genes to virulence in different infection models has been explored using *SAP*-null mutant strains [75]. Gene disruption approaches previously proved that Δ*SAP1*, Δ*SAP2*, and Δ*SAP3* null mutants exhibited attenuated virulence in models of acute systemic candidiasis [76]. Moreover, the transcriptional responses of Saps during human mucosal infections in vitro and in vivo have already illustrated that the *SAP* gene family, as a whole, is responsible for the damage-inducing ability of *C. albicans* [77].

The capacity of pathogenic fungi to cause mycosis with a variety of clinical manifestations depends on the complex interactions between the fungus and the human host, with adhesion proteins playing a crucial role as virulence factors in many cases [78]. Consequently, these molecules have become important targets for studying yeast biology and the development of new therapeutic approaches for treating fungal infections and designing novel drugs [79,80,81,82].

## 4. Conclusions

Taken together, all the conducted experiments in this study allowed us to demonstrate that the ability of *C. albicans* to produce Saps1–3 antigens can be modulated by soluble proteins and their concentrations, as well as by the interaction with different cell lineages and during the in vivo infectious process. Given that Saps represent a major virulence attribute utilized by many *Candida* species during the infectious process, our intention was to highlight how the modulation of this specific factor is essential for the success and survival of the yeast in the hostile environment of the human body.

## Figures and Tables

**Table 1:** Effect of different soluble proteins on the expression of *Candida albicans* Saps1–3 antigens.

YCB Medium +	% Fluorescent Cells	Mean of Fluorescence Intensity
**Bovine serum albumin**	29.3 ± 2.5	50.9 ± 1.4
**Immunoglobulin G**	11.6 ± 1.9 *	59.0 ± 0.4
**Total bovine serum**	15.0 ± 3.3 *	29.3 ± 8.3 **
**Total human serum**	18.1 ± 3.2 *	28.2 ± 6.1 **
**Gelatin**	19.1 ± 2.4 *	89.0 ± 0.6 **
**Laminin**	40.5 ± 0.6 *	48.5 ± 0.6
**Human serum albumin**	40.6 ± 1.2 *	97.1 ± 0.2 **
**Fibrinogen**	43.7 ± 1.3 *	62.4 ± 0.6
**Hemoglobin**	49.5 ± 2.0 *	144.5 ± 6.4 **
**Mucin**	50.2 ± 1.3 *	118.4 ± 2.4 **

The symbols represent the systems that were statistically different compared to BSA control (* *p* < 0.05; ** *p* < 0.001 by one-way ANOVA).

**Table 2:** Effect of in vitro interaction of *Candida albicans* yeast cells with animal cells and in vivo infection of immunosuppressed BALB/c mice on the expression of Saps1–3 antigens.

Systems	% Fluorescent Cells	Mean of Fluorescence Intensity
**YCB-BSA**	29.3 ± 2.5	50.9 ± 1.4
**Epithelial cells (HEp-2)**	78.0 ± 4.3 *	152.1 ± 21.5 **
**Murine macrophage cells**	82.7 ± 2.1 *	178.2 ± 10.8 **
**Kidney (mouse infection)**	95.5 ± 3.6 **	246.6 ± 25.5 **

The symbols represent the systems that were statistically different compared to YCB-BSA control (* *p* < 0.05; ** *p* < 0.001 by one-way ANOVA).

## Data Availability

Data are contained within the article.
